# The natural FGF-trap long pentraxin 3 inhibits lymphangiogenesis and lymphatic dissemination

**DOI:** 10.1186/s40164-022-00330-w

**Published:** 2022-11-01

**Authors:** Marta Turati, Arianna Giacomini, Sara Rezzola, Federica Maccarinelli, Giorgia Gazzaroli, Sonia Valentino, Barbara Bottazzi, Marco Presta, Roberto Ronca

**Affiliations:** 1 Department of Molecular and Translational Medicine, University of Brescia, viale Europa 11, 25123 Brescia, Italy; 2 IRCCS Humanitas Research Hospital, Milan, Italy

**Keywords:** Long pentraxin 3, Lymphangiogenesis, Fibroblast growth factor, Melanoma, Metastasis

## Abstract

The lymphatic vascular system represents a major route for dissemination of several solid tumors, including melanoma. Even though the members of the Vascular Endothelial Growth Factor family VEGF-C and VEGF-A have been shown to drive tumor lymphangiogenesis, experimental evidence indicates that also the pro-angiogenic factor Fibroblast Growth Factor-2 (FGF2) may play a role in the lymphangiogenic switch by triggering the activation of lymphatic endothelial cells (LECs) in cooperation with VEGFs.

The soluble pattern recognition receptor Long Pentraxin 3 (PTX3) acts as a natural FGF trap, thus exerting an oncosuppressive role in FGF-dependent tumors. Here, the capacity of PTX3 to modulate lymphangiogenesis was assessed in vitro and in vivo. The results demonstrate that recombinant human PTX3 inhibits the lymphangiogenic activity exerted by the VEGF-A/FGF2/sphingosine-1-phosphate (VFS) cocktail on human and murine LECs. In keeping with in vitro data, a reduced lymphangiogenic response was observed in a lymphangiogenic Matrigel plug assay following the subcutaneous injection of the VFS cocktail in PTX3-overexpressing transgenic TgN(Tie2-hPTX3) mice when compared to wild-type or *Ptx3* null animals. Accordingly, the capacity of B16F10-VEGFC-luc melanoma cells to colonize the primary tumor-draining lymph node after grafting into the foot pad was dramatically impaired in PTX3-overexpressing mice.

Together with the observation that both the VFS cocktail and melanoma cell conditioned media caused a significant downregulation of PTX3 expression in LECs, these data indicate that the FGF trap activity of PTX3 may exert a key effect in the modulation of lymphangiogenesis and tumor metastatic dissemination.

**Supplementary Information:**

The online version contains supplementary material available at 10.1186/s40164-022-00330-w.

To the Editor,

In cancer, the lymphatic vascular system represents a major route for the dissemination of solid tumors. During tumor progression, cancer cells enter the tumor-draining LNs, which represent the first site of metastasis, and then reach distant organs [1].

I this context, the lymphangiogenic switch is induced and sustained by tumor-derived pro-lymphangiogenic factors, including the Vascular Endothelial Growth Factor family members VEGF-C and VEGF-A [1–3], nevertheless scattered pieces of evidence indicate that also the pro-angiogenic Fibroblast Growth Factor-2 (FGF2) may play a relevant role in activating lymphangiogenesis through different mechanisms. Indeed, FGF2 has been shown to bind FGF Receptor 3 (FGFR3) expressed by lymphatic endothelial cells (LECs) [4] and to interact with the Lymphatic Vessel Endothelial hyaluronic acid receptor 1 (LYVE-1) which participates in FGF2 internalization [5]. In addition, FGF2 has been proposed to cooperate with VEGF-C and trigger tumor lymphangiogenesis and metastasis through FGFR1/VEGFR3-dependent pathways [6].

The soluble pattern recognition receptor Long Pentraxin 3 (PTX3) is a member of the pentraxin family locally produced by different cell types in response to inflammatory signals, and exerting pleiotropic functions both in physiological and pathological conditions, including cancer [7]. In the tumor microenvironment, PTX3 has been shown to play pro- or anti-tumor activities depending on cancer type. In particular, the oncosuppressive role of PTX3 relies on its anti-inflammatory properties [8] and on the capacity to act as a FGF trap, thus preventing FGF-dependent tumor cell survival and proliferation, as well as angiogenesis and epithelial-to-mesenchymal transition [9–11]. Despite several observations about the impact of PTX3 on various aspects of cancer progression, to date no data are available about the role of PTX3 in lymphangiogenesis and tumor lymphogenous dissemination.

It has been shown that the combination of VEGF-A, sphingosine-1-phosphate (S1P) and FGF2 represents a potent pro-lymphangiogenic cocktail (VFS) able to induce LEC activation in vitro and lymphangiogenesis in vivo [12]. Notably, none of these mediators is able to induce the proliferation and 3D-sprouting of human dermal lymphatic endothelial cells (HDLECs) when tested alone or in double combination, thus indicating that the lymphangiogenic activity depends upon the synergistic action of all the components, including FGF2 (**Fig. S1**). On this basis, to assess the impact of the natural FGF-trap protein PTX3 on lymphangiogenesis, murine lymphatic endothelial MELC-2 cells and human HDLECs were treated with the VFS cocktail in the absence or in the presence of recombinant human PTX3 (rhPTX3). In keeping with its FGF-trap activity, rhPTX3 reduced the activation of FGFRs in both murine and human LECs stimulated with VFS (Fig. 1 A). Accordingly, in both MELC-2 and HDLEC cells stimulated with VFS, treatment with rhPXT3 resulted in a significant inhibition of proliferation, migration, 3D-sprouting and tube formation activity (Fig. 1B-D). Accordingly, a similar inhibitory effect was observed when HDLECs were treated with VFS in the presence of soluble FGFR1 (**Fig. S2A**), with no effect observed by the short pentraxin serum amyloid P component (SAP) which is devoid of any FGF-antagonist activity (**Fig. S2B**).

**Fig. 1:**
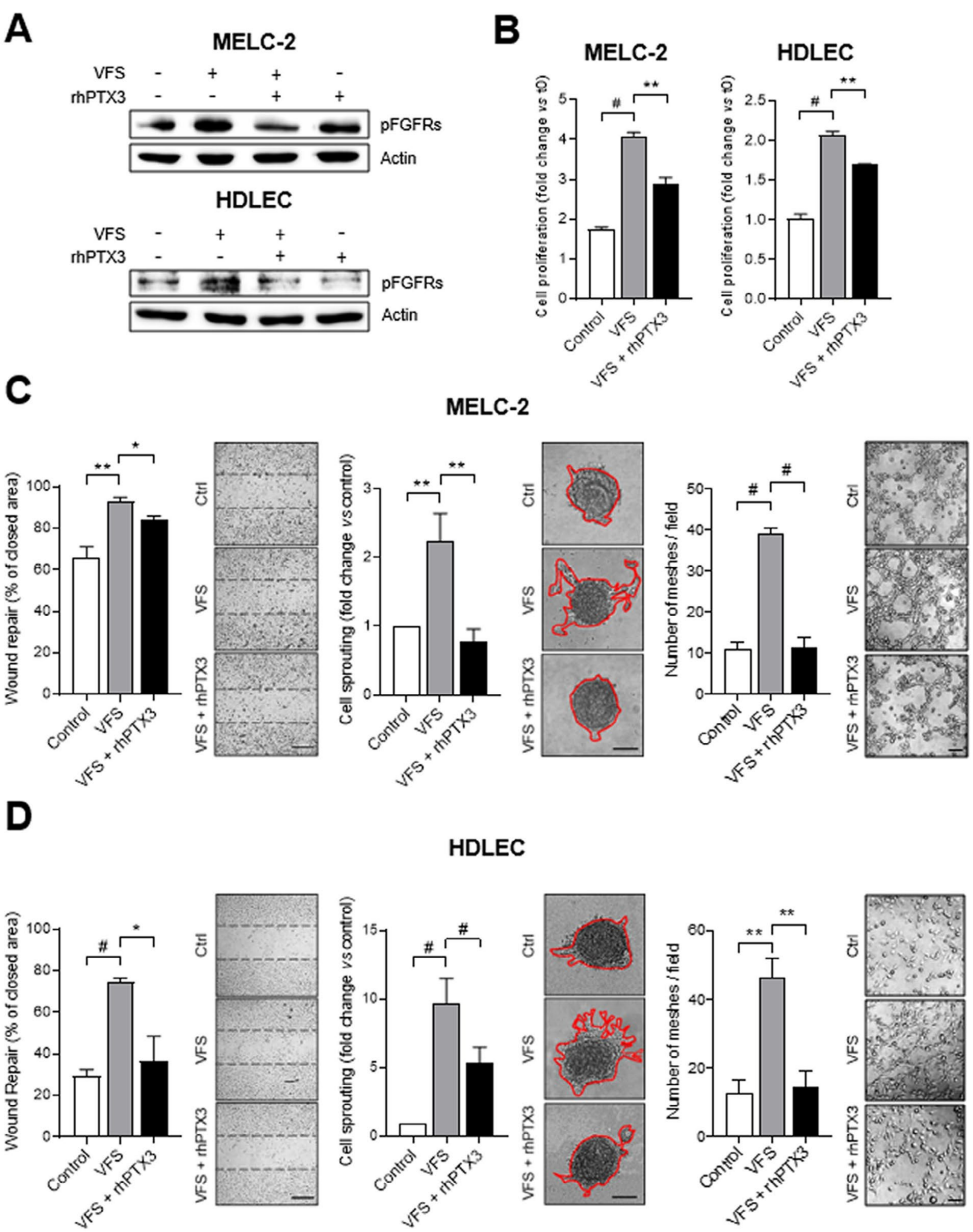
**PTX3 modulates the VFS-mediated LEC activation in vitro. A**) Western blot analysis of MELC-2 (upper panel) and HDLEC (lower panel) treated or not with VFS mixture and rhPTX3. **B**-**D**) The effect of treatment with VFS on MELC-2 (**C**) and HDLEC (**D**) in presence or in absence of rhPTX3 was evaluated in terms of cell proliferation, cell motility in wound healing assay, sprout formation and tube formation. Scale bars: 50 μm. Data are expressed as mean ± SEM, experiments were performed in triplicate. *p < 0.05, **p < 0.01, #p < 0.001

Notably, treatment with VFS caused a significant downregulation of PTX3 both at mRNA (Fig. 2 A) and protein (Fig. 2B) level. Similar results were obtained when HDLECs were treated with the conditioned medium obtained from human (A375) and murine (B16F10) melanoma cells (Fig. 2 C), thus suggesting that PTX3 modulation might represent a requirement in the activation of LECs during tumor lymphangiogenesis.

**Fig. 2:**
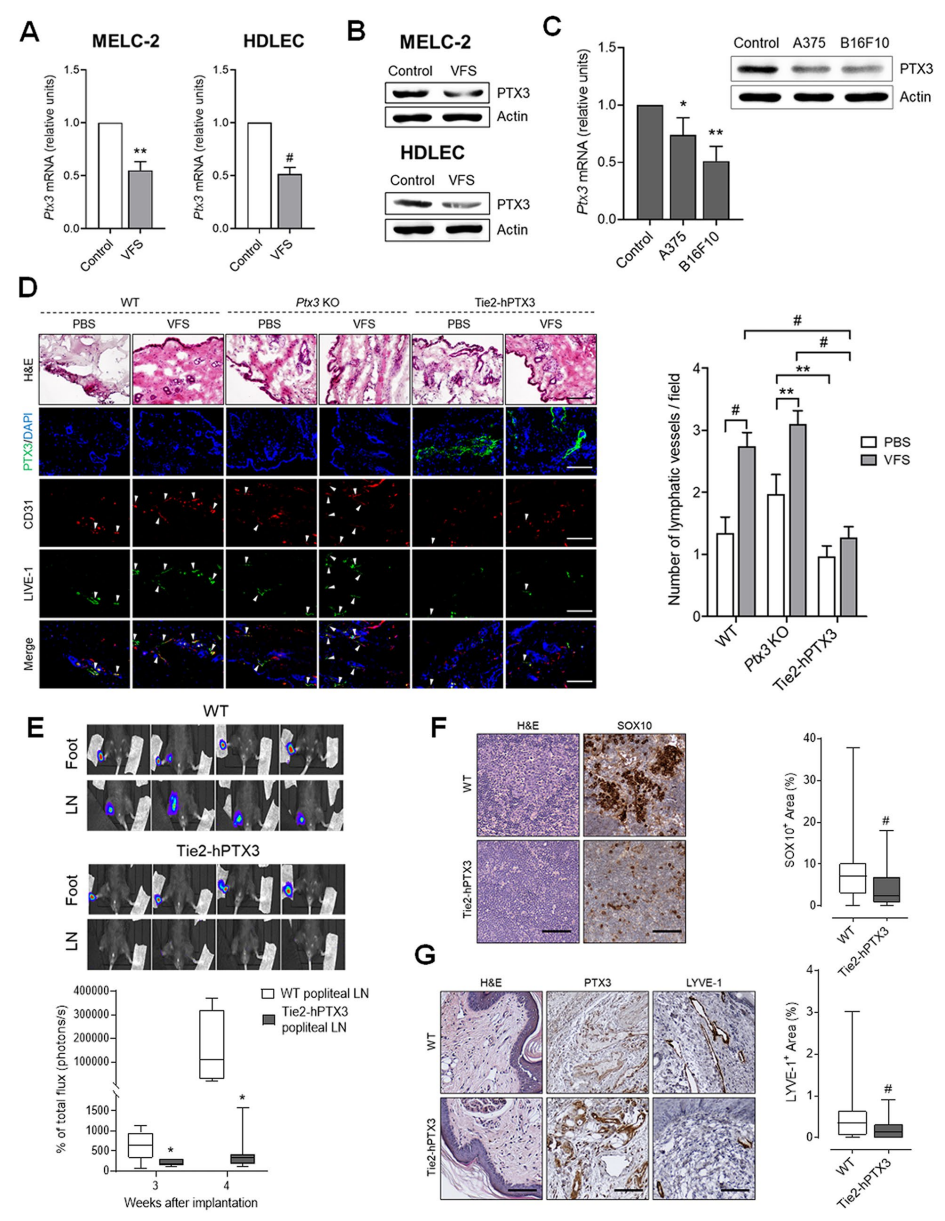
**PTX3 impairs the lymphangiogenesis and lymphatic dissemination.** qPCR of PTX3 mRNA (**A**) and Western blot analysis (**B**) of MELC-2 and HDLEC treated with VFS or not (control). **C**) qPCR (left panel) and Western blot analysis (right panel) for PTX3 expression in HDLEC treated with conditioned media from melanoma cells. **D**) Representative images of hematoxylin and eosin (H&E) and immunofluorescence staining on cryosections obtained from WT, *Ptx3*^*−/−*^ (*Ptx3* KO) and TgN(Tie2-hPTX3) mice implanted with Matrigel plugs containing PBS or VFS cocktail. PTX3 expression is detected in green by anti-PTX3 staining and newly formed LVs (white arrows) in the dermis overlying the implanted Matrigel plug were identified through immunofluorescence double staining using anti-LYVE-1 (green) and anti-CD31 (red) antibodies. The number of LVs per field was quantified. At least 37 fields for each experimental point were analyzed, data are expressed as mean ± SEM. **E**) WT and TgN(Tie2-hPTX3) mice were injected with B16F10-VEGFC-luc cells in the foot pad and bioluminescence imaging was performed 3 and 4 weeks after injection (n = 8 mice/group). Top panel: representative pictures of feet and LN bioluminescence at 4 weeks are reported. **F**) LNs from WT and TgN(Tie2-hPTX3) mice injected with B16F10-VEGFC-luc cells were stained and SOX10^+^ areas quantified. **G**) H&E and IHC of the foot pad dermis in proximity to the primary tumor of WT and TgN(Tie2-hPTX3) mice injected with B16F10-VEGFC-luc cells. Representative pictures (left panel) and quantification (right panel) are reported. Data are the mean ± SEM. Data expressed in box-and-whisker plot, represent the 25th to the 75th percentiles, lines indicate the median values, and whiskers indicate the range of values. *p < 0.05, #p < 0.001. Scale bars: 50 μm

These data point to a role for PTX3 in lymphangiogenesis and demonstrate that its capacity to inhibit the FGF2/FGFR system hampers the acquisition of a pro-lymphangiogenic phenotype in LECs in vitro.

In order to extend these observations in vivo, a lymphangiogenic Matrigel plug assay was performed [12] in which the VFS cocktail was injected subcutaneously in C57BL/6 wild-type (WT), in *Ptx3* null (KO) mice or in transgenic TgN(Tie2-hPTX3) mice characterized by endothelial overexpression and stromal accumulation of PTX3 [10].

As shown in Fig. 2D, after 3 weeks, the quantification of newly formed CD31^+^/LYVE-1^+^ lymphatic vessels (LVs) in the dermis overlaying the implanted Matrigel revealed that VFS triggered a significant increase of newly formed LVs in both WT and KO mice when compared to control/PBS plugs. At variance, no significant lymphangiogenic response was induced in PTX3 overexpressing TgN(Tie2-hPTX3) animals, thus indicating that FGF trapping by endogenous PTX3 is able to suppress lymphangiogenesis in vivo.

Given the anti-lymphangiogenic potential of PTX3 in vitro and in vivo, and the modulatory effect exerted by VFS and melanoma cell conditioned medium on PTX3 expression in LECs, we explored the impact exerted by PTX3 on lymphatic dissemination of melanoma cells in vivo taking advantage of the B16F10-VEGFC-luc melanoma cell line. This model is characterized by an extremely efficient lymphatic dissemination and the capacity to colonize the primary tumor-draining LN after injection into the foot pad [13]. B16F10-VEGFC-luc cells were injected into the foot pad of WT and PTX3 overexpressing TgN(Tie2-hPTX3) mice and cancer cells dissemination was monitored by bioluminescence imaging (Fig. 2E). As shown in Fig. 2E, in vivo imaging performed at 3 and 4 weeks after tumor injection revealed that PTX3 overexpression significantly reduced the metastatic spreading of B16F10-VEGFC-luc cells and their capacity to colonize the tumor-draining popliteal LN in TgN(Tie2-hPTX3) mice when compared to WT animals. This was further confirmed by melanoma cells staining (SOX10^+^ areas) in the LN at week 4 (Fig. 2 F). In line with this result, immunohistochemical analysis of the foot pad dermis in proximity to the primary tumor revealed a significant decrease of LYVE-1^+^ LVs that goes along with the high levels of PTX3 in TgN(Tie2-hPTX3) mice when compared to WT animals (Fig. 2G).

It is widely recognized that a balance between angiogenesis inducers and inhibitors controls the rate of new blood vessel formation [14]. Our data indicate for the first time that PTX3 plays a negative regulatory role in LEC response to pro-lymphangiogenic stimuli and that its downregulation by melanoma cells may represent a mechanism to overcome this inhibitory activity to sustain lymphangiogenesis and foster tumor cell dissemination. A better understanding of these regulatory mechanisms will add new information about the biology of lymphatic vessels and may pave the way to focused therapeutic approaches aimed to reduce lymphatic metastatic dissemination.

## Electronic supplementary material

Below is the link to the electronic supplementary material.

Supplementay Figures and methods

## Data Availability

All data are included in the study and available from the corresponding author on reasonable request.
