# Supplementary material for: The natural FGF-trap long pentraxin 3 inhibits lymphangiogenesis and lymphatic dissemination
Source: Exp Hematol Oncol. 2022 Nov 1;11:84. doi: 10.1186/s40164-022-00330-w (PMC9623950; doi:10.1186/s40164-022-00330-w)
Supplement: Supplementary file 1 — Supplementay Figures and methods [file 40164_2022_330_MOESM1_ESM.pdf]

SUPPLEMENTARY FIGURES

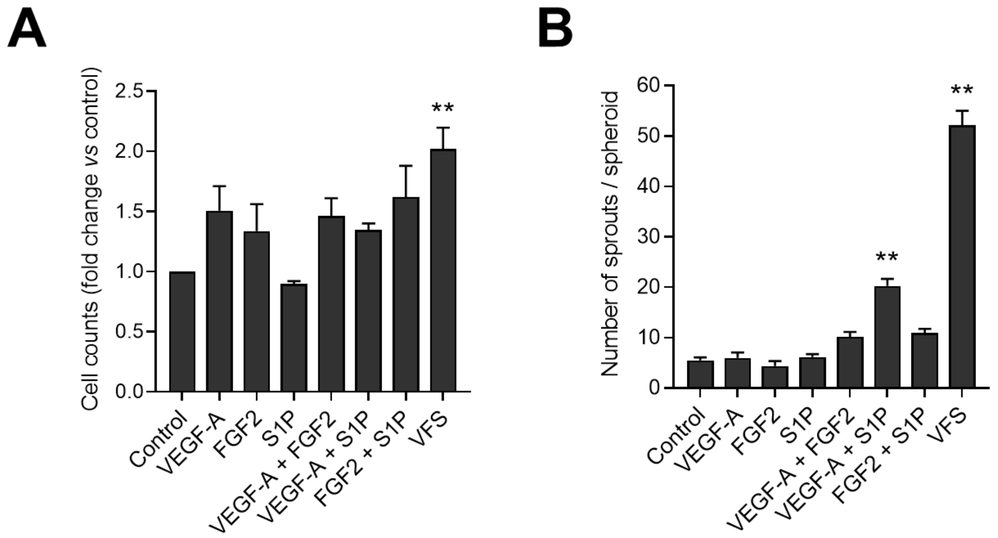

Figure S1. Treatment with VFS significantly promotes cell proliferation and sprout formation in HDELCs. A) Cell counts of HDLECs treated or not (control) with VEGF-A, FGF2 and S1P alone or in combination. B) Number of sprouts per HDLEC spheroid treated or not (control) with VEGF-A, FGF2 and S1P alone or in combination. Data are expressed as mean ± SEM; **p < 0.01.

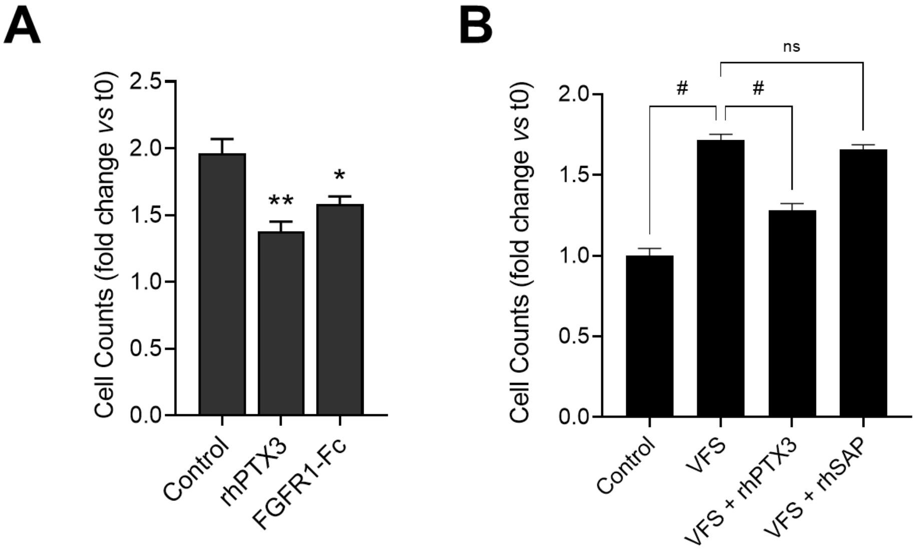

Figure S2.

A) Treatment with soluble recombinant human PTX3 (rhPTX3) and recombinant FGFR1 (FGFR1-Fc) hampers cell proliferation in VFS-treated HDLECs. B) Contrary to rhPTX3, treatment with recombinant human SAP (rhSAP) has no effect on the VFS-induced proliferation of HDLECs. *p < 0.05, **p < 0.01, #p < 0.001, ns= not significant.

SUPPLEMENTARY MATERIALS AND METHODS

Reagents. DMEM and RPMI culture media, trypsin, glutamine, non-essential amino acids, Na pyruvate and geneticin were purchase from GIBCO Life Technologies (Grand Island, NY). EGM-2 and EBM media were from Lonza (Walkersville, MD USA). Fetal bovine serum (FBS) was provided by Euroclone (Milan, Italy). Porcine heparin was obtained from Sigma-Aldrich (St.Louis, MO USA). Endothelial cell growth supplement (ECGS), Millex-GP 0.22 µm syringe filter unit and Amicon centrifuge tubes were from Merck Millipore (Darmstadt, Germany). Recombinant human VEGF (VEGF-A_{165} isoform) was kindly provided by K. Ballmer-Hofer (PSI, Villigen, Switzerland). Recombinant human FGF2 was obtained from Tecnogen (Caserta, Italy). S1P was purchased from Avanti Polar Lipids Inc (Alabaster, USA). QIAzol lysis reagent were provided by QIAGEN (Germantown, MD USA). Bio-Rad protein assay dye reagent concentrate and Clarity western ECL substrate were purchased from Bio-Rad Laboratories (Hercules, CA USA). M-MLV reverse transcriptase and random primers were from Invitrogen Life technologies (Carlsbad, CA USA). dNTPs and rabbit anti-FAK polyclonal antibody were from Thermo fisher scientific (Waltham, MA USA). DNAse I recombinant Rnase free was from Promega (Madison, WI USA). Rabbit anti-PTX3 polyclonal antibody was kindly provided by B. Bottazzi (Humanitas Clinical Institute, Milan, Italy). Mouse LYVE-1 biotinylated antibody was from R&D Systems (Minneapolis, MN USA). Mouse monoclonal anti-SOX10 antibody was provided by Santa Cruz Biotechnology (Dallas, TX USA). Rat anti-CD31 monoclonal antibody was purchased from BD Pharmingen (San Diego, CA USA). Rabbit anti-LYVE1 polyclonal antibody was purchased from AngioBio (San Diego, CA USA). Vectashield mounting medium was purchased from Vector Laboratories (Burlingame, CA USA). Optimal cutting temperature compound (OCT) was from Bio-optica (Milan, Italy). Vectastain Elite ABC kit was from Vector Laboratories (Burlingame, CA,USA). Goat Anti-Mouse IgG (HRP polymer) and Goat Anti-Rabbit IgG (HRP polymer) were purchased from Abcam (Cambridge, UK). 3,3′-Diaminobenzidine (DAB) was from Roche (Monza, Italy). DPX Mountant for histology was provided by Sigma-Aldrich (St.Louis, MO USA).

Cell cultures. MELC-2 were grown on plastic surface coated with 0.1% porcine gelatin in DMEM medium supplemented with 10% heat-inactivated FBS, 10% conditioned medium from S180 cells (cultured in DMEM supplemented with 10% heat-inactivated FBS), 1.0 mM glutamine, 1.0 % non-essential amino acids, 1.0 mM Na pyruvate, 100 µg/ml porcine heparin and 30 µg/ml ECGS. HDLECs (Promocell) were maintained in EGM-2 medium. Human melanoma A375 cells were cultured in DMEM supplemented with 10% FBS. Murine melanoma B16F10 cells were maintained in RPMI medium supplemented with 10% FBS. The VEGF-C/luciferase expressing B16F10-VEGF-

C-*luc* cells were cultured in DMEM supplemented with 10% FBS in the presence of 1.6 mg/ml geneticin [25].

The conditioned media (CM) were obtained from the melanoma cell lines by starving them for 48 hours in FBS-free medium. The CM were collected, centrifuged to remove remaining cells or cellular debris, and filtered with 0.22 µm syringe filter. Then, CM were concentrated (10x) using Amicon centrifuge tubes with a molecular weight cut-off of 10 KDa. Concentrated CM were stored at -20 °C.

Cell proliferation assay. HDLEC were treated in EBM plus 5% FBS in the presence of 40 ng/ml VEGF-A, 40 ng/ml FGF2 and 2 µM S1P alone or in different combinations with or without 150 nM PTX3. MELC-2 were treated in DMEM plus 1% FBS with the lymphangiogenic cocktail composed by 40 ng/ml FGF2, 40 ng/ml VEGF-A and 2 µM S1P (VFS) in the absence or in the presence of 150 nM PTX3. After 48 hours treated cells were detached through trypsinization and counted using the MACSQuant Analyzer (Miltenyi Biotec).

Spheroid sprouting assay. HDLEC and MELC-2 spheroids were prepared in 20% methylcellulose medium and embedded in fibrin gel. HDLEC spheroids were treated with FGF2, VEGF-A and S1P alone and in different combinations. HDLEC and MELC-2 spheroids were stimulated with VFS in the absence or in the presence of 150 nM PTX3. After 24 hours of treatment sprouts were counted and photographed.

Wound healing assay. MELC-2 and HDLEC monolayers were scraped with a 200 µl tip and maintained in culture medium plus 1% and 2.5% FBS, respectively, in the presence of VFS with or without 150 nM PTX3. After 24/36 hours pictures of the wounded monolayers were acquired and cell migration was quantified using ImageJ software.

Tube formation assay. MELC-2 and HDLEC cells (2.0 x 10^5) were seeded in 48-well plate pre-coated with matrigel (Cultrex BME) in culture medium plus 5% FBS, in the presence of VFS with or without 150 nM PTX3. After 3 or 4 hours for HDLEC and MELC-2, respectively, images were acquired and the number of meshes was quantified using ImageJ software.

Real Time PCR analysis. MELC-2 and HDLEC were treated in culture medium 1% FBS for 12/24 hours with VFS. HDLEC cells were treated with melanoma cell conditioned medium (10x) diluted 1x in basal culture medium EBM, supplemented with 2.5% FBS. Treated cells were processed for mRNA expression analysis. Total RNA was extracted using QIAzol reagent according to manufacturer's instructions. After contaminating DNA digestion using DNAse, 2 µg of total RNA were retro transcribed with MMLV reverse transcriptase using random hexaprimers in a final volume of 20 µl. cDNA was diluted 1:2 and then analyzed by quantitative PCR using primers specific for

human or murine PTX3 mRNA detection (hPTX3: Forward primer: 5'-CATCTCCTTGCGATTCTGTTTTG-3'; reverse primer: 5'-CCCATTCCGAGTGCTCCTGA-3'. mPTX3: Forward primer: 5'- GACCTCGGATGACTACGAG-3'; reverse primer: 5'-CTCCGAGTGCTCCTGGCG-3'). Housekeeping gene human GAPDH and murine β-actin mRNA was detected for normalization using specific primers (hGAPDH: Forward primer: 5'-GAAGGTCGGAGTCAACGGATT-3'; reverse primer: 5'-TGACGGTGCCATGGAATTTG-3'. mβ-actin: Forward primer: 5'-GTGACGAGGCCCAGAGCAAGAG-3'; reverse primer: 5'-AGTCCATCACAATGCCCGTGGT-3').

Western Blot analysis. MELC-2 and HDLEC were treated in culture medium plus 1% and 2.5% FBS, respectively, with VFS for 24 hours and then lysed with lysis buffer (TRIS-HCl pH 7 50mM, NaCl 150mM, Triton X-100 1%, BriJ 0.1%). Protein concentrations were determined using the Bradford protein assay. 30 μg protein/sample were separated by SDS-PAGE and the amount of PTX3 was analyzed through western blot analysis, using rabbit anti-PTX3 antibody and rabbit anti-FAK antibody for normalization. Primary antibodies detection was performed using HRP-conjugated secondary antibodies incubated with Clarity western ECL substrate before chemiluminescence detection through ChemiDoc (Bio-Rad).

***In vivo* experiments.** Animal experiments were approved by the local animal ethics committee (OPBA, Organismo Preposto al Benessere degli Animali, Università degli Studi di Brescia, Italy) and were performed in accordance with national guidelines and regulations. TgN(Tie2-hPTX3) mice were obtained as reported in [18] and are characterized by human PTX3 overexpression under the control of the endothelial specific Tie2/Tek transcription regulatory sequences and by its accumulation in the perivascular and stromal microenvironment. *Ptx3*$^{-/-}$ mice were provided by C. Garlanda (Humanitas Clinical Institute, Milan, Italy) and are characterized by *PTX3* knockout [23].

Matrigel plug assay. Wild-type (WT), *Ptx3*$^{-/-}$ and TgN(Tie2-hPTX3) C57BL/6 mice were injected subcutaneously with 400 μl of Matrigel containing phosphate-buffered saline (PBS) or VFS cocktail composed by VEGF-A500 ng/ml, FGF2 500 ng/ml and S1P 2 μM. After 21 days, the matrigel plugs together with the overlying skin were removed, embedded in OCT and frozen. Five μm thick cryosections were processed, double-immunostained with rat anti-CD31 and rabbit anti-LYVE1 antibodies, and then mounted with Vectashield mounting medium containing DAPI for nuclear staining. Pictures were taken with a 20x magnification using Axiovert 200M and AxioCam MRm (Zeiss), and LYVE-1/CD31 double positive LVs were counted.

Footpad subcutaneous assay. 200000 B16F10-VEGF-C-*luc* cells in a volume of 10 μl were injected in the foot pad of WT and transgenic TgN(Tie2-hPTX3) C57BL/6 mice. Bioluminescence imaging

was performed 3 and 4 weeks after implantation to monitored B16F10-VEGF-C-*luc* cells dissemination to the draining popliteal LN using IVIS Lumina III.

Statistical analyses. Statistical analyses were performed exploiting the statistical package Prism 8 (GraphPad Software). Statistical significance was evaluated using unpaired two-samples t-test (*p < 0.05; **p < 0.01; #p < 0.001).
